# Encapsulants Affect Liposome Surface Interactions with Biological Systems

**DOI:** 10.1002/smll.202505312

**Published:** 2025-06-19

**Authors:** Clemens Spitzenberg, Christoph Bruckschlegel, Ferdinand Holzhausen, Sebastian Boesl‐Bichlmeier, Coralie Pasquier, Patrick Nuernberger, Pierre Bauduin, Antje J. Baeumner

**Affiliations:** ^1^ Institut für Analytische Chemie Chemo‐ und Biosensorik Universität Regensburg D‐93053 Regensburg Germany; ^2^ Institut für Physikalische und Theoretische Chemie Universität Regensburg D‐93053 Regensburg Germany; ^3^ ICSM CEA CNRS ENSCM University Montpellier Bagnols sur Cèze F‐30207 France

**Keywords:** complement assay, fluorescence, liposomes, SAXS, spectroscopy

## Abstract

Liposomes are self‐assembled lipid bilayer nanostructures with an inner aqueous core used for diagnostic signal amplification, drug delivery, and as biomimics. Small molecules and proteins are typically encapsulated. While the lipid composition is used to control liposome and surface characteristics, overlooked is the effect entrapped molecules may have on the outer surface through interface activity. Here, it is demonstrated how different dyes not only distribute between the aqueous core and bilayer but also significantly affect the outer surface chemistry thus influencing interactions with reaction partners and sample matrices. Specifically, IR‐783, sulforhodamine B (SRB), and 1,3,6,8‐pyrenetetrasulfonic acid (PTSA) are encapsulated in liposomes of standard bioanalytical composition. Spectroscopic and small‐angle X‐ray scattering data indicate interactions of IR‐783 with the liposome membrane and its strong influence on the bilayer structure. Increasing SRB concentrations show potential adsorption at liposome bilayer surfaces, whereas PTSA does not interact with the bilayer itself. Surprising is the correlating effect on biological systems discovered through the complement system as a model. Liposomes incubated with serum reveal complement protein interaction with the liposome surface depending on the dye and not only on the lipid composition. This study emphasizes the need for careful selection of both lipid and encapsulant formulations in any biological application.

## Graphical abstract

This study demonstrates how dyes encapsulated in liposomes, lipid bilayer nanostructures with an inner aqueous core, affect liposome surface chemistry and interactions with biological matrices. This is achieved by investigating the effects of dye interactions with liposomal membranes using spectroscopy, small‐angle X‐ray scattering, and a bioassay. Results emphasize the need for careful selection of both lipids and encapsulants in bioanalysis.

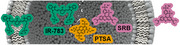

## 1 Introduction

Liposomes, first described as swollen phospholipid systems by Bangham et al. in 1965,^[^ ^1^ ^]^ have been established as a powerful and versatile tool in modern diagnostics and research. The simplest form of liposomes has been described as a self‐assembled structure containing an inner aqueous cavity surrounded by a lipid bilayer.^[^ ^2^ ^]^ The bilayer is composed predominantly of phospholipids with diversity in the chemical structure and characteristics of their head group and the carbon chain length of their hydrophobic tail. Phosphatidylcholine, phosphatidylethanolamine, phosphatidylserine, and phosphatidylglycerol are the most abundant naturally occurring head groups in biological systems and are also applied in liposome preparation.^[^ ^3^ ^]^ Increased stability is often achieved by including cholesterol in the lipid composition causing tighter packing of the phospholipids, thereby reducing bilayer permeability and forming more rigid liposomes.^[^ ^4^ ^]^ Liposomes can be prepared in various sizes, ranging from small vesicles of 25–50 nm to vesicles up to a few millimeters in size, depending on the number of bilayer shells (i.e. the lamellarity), the lipid composition, the preparation procedure, and the molecules inside the aqueous compartment.^[^ ^5^ ^]^ Already in the 1970s, scientists began to explore the use of liposomes as drug or biomolecule carriers.^[^ ^6^ ^]^ Since then, the cavity has been demonstrated to be suitable for encapsulation of a variety of hydrophilic molecules, including fluorescent dyes,^[^ ^7^ ^]^ electroactive species,^[^ ^8^ ^]^ oligonucleotides,^[^ ^9^ ^]^ drugs,^[^ ^10^ ^]^ or enzymes.^[^ ^11^ ^]^ Overall, this flexibility in lipid composition, surface charge, and modification, liposome size, and encapsulant allows for wide versatility in the application of liposomes. These range from signal amplification,^[^ ^12^ ^]^ the use as biomimics of cells or other biological vesicles in bioassays, and to the application as drug delivery vesicles in therapeutic contexts.^[^ ^13^ ^]^ However, the introduction of liposomes into biofluids and biological systems is affected by corona formation, opsonization, or more general deposition of biomolecules on the surface.^[^ ^14^ ^]^ These reactions to the intruding nanomaterial alter the surface characteristics, which often also hampers their efficacy due to the accelerated clearance rates of such materials.

Small‐angle X‐ray scattering (SAXS) is a technique used to study such structural and physical properties and their changes of nanomaterials. The method is based on the irradiation of samples with monochromatic, collimated X‐rays. The X‐rays are scattered depending on the electron density characteristics of the nanomaterial and are subsequently recorded with a 2D‐detector.^[^ ^15^ ^]^ The intensity of the scattered X‐rays as a function of the scattering vector q (q  =  4π/λ sin(θ/2); with the wavelength of X‐rays λ and scattering angle θ) provide information about the form factor (revealing size, shape, and size distribution) and the structure factor (revealing structural organization and particle interactions) of the nanomaterial.^[^ ^16^ ^]^ The scattered X‐rays are typically investigated in a range from q  =  10^−1^ – 10^1^ nm^−1^, corresponding to structures in the range of ≈1–100 nm, making SAXS a powerful technique for the study of nanomaterials. While smaller angles (lower q values) are associated with larger features, larger angles (higher q values) refer to smaller structures, as can be seen from the relation d = 2π/q where d is the distance probed in real space at a given q value.^[^ ^15^ ^]^ Especially the lipid bilayer of liposomes, with a thickness of roughly 5 nm and with a defined electron density profile, is well‐suited for study by SAXS, as binding or accumulation of molecules (including dyes) on the bilayer will lead to changes in the scattering.^[^ ^17^, ^18^, ^19^ ^]^

Due to the frequent application of liposomes in biofluids, most current research focuses on surface characteristics and modifications to circumvent unwanted reactions of biological systems with liposomes. For instance, PEGylation is often mentioned as a suitable surface modification as well as a neutral surface charge to prevent protein deposition.^[^ ^14^, ^20^, ^21^ ^]^ The broad application of PEGylation in pharmaceutical or cosmetical formulations induces anti‐PEG antibody production in many individuals, which triggers unwanted immune reactions (e.g. faster clearance of pharmaceuticals) as shown in recent studies.^[^ ^22^, ^23^ ^]^ However, in this work, rather than intensifying further optimizations on the liposome surface via lipid compositions or modifications, our goal was to investigate the relevance of effects entrapped molecules may have on the lipid bilayer and the characteristics of the outer surface. This has received little attention to date, yet it represents a significant factor which potentially influences the reactivity of liposomes. In the case of liposomes, the effect entrapped molecules may have on the lipid bilayer and the characteristics of the outer surface has received little attention to date. Therefore, this work focuses on examining how different fluorescent dyes can affect the liposome surface chemistry and, through this influence, interactions with biological systems using the human complement system as an example. Specifically, the fluorophores IR‐783, sulforhodamine B (SRB), and 1,3,6,8‐pyrenetetrasulfonic acid (PTSA) were encapsulated in liposomes. These were then analyzed for their size, surface charge, and spectroscopic characteristics, and SAXS measurements were performed to determine changes in the lipid bilayer structure. Finally, the effect of the different liposomes on the human complement system was investigated in serum samples.

## 2 Results and Discussion

**Figure 1:**
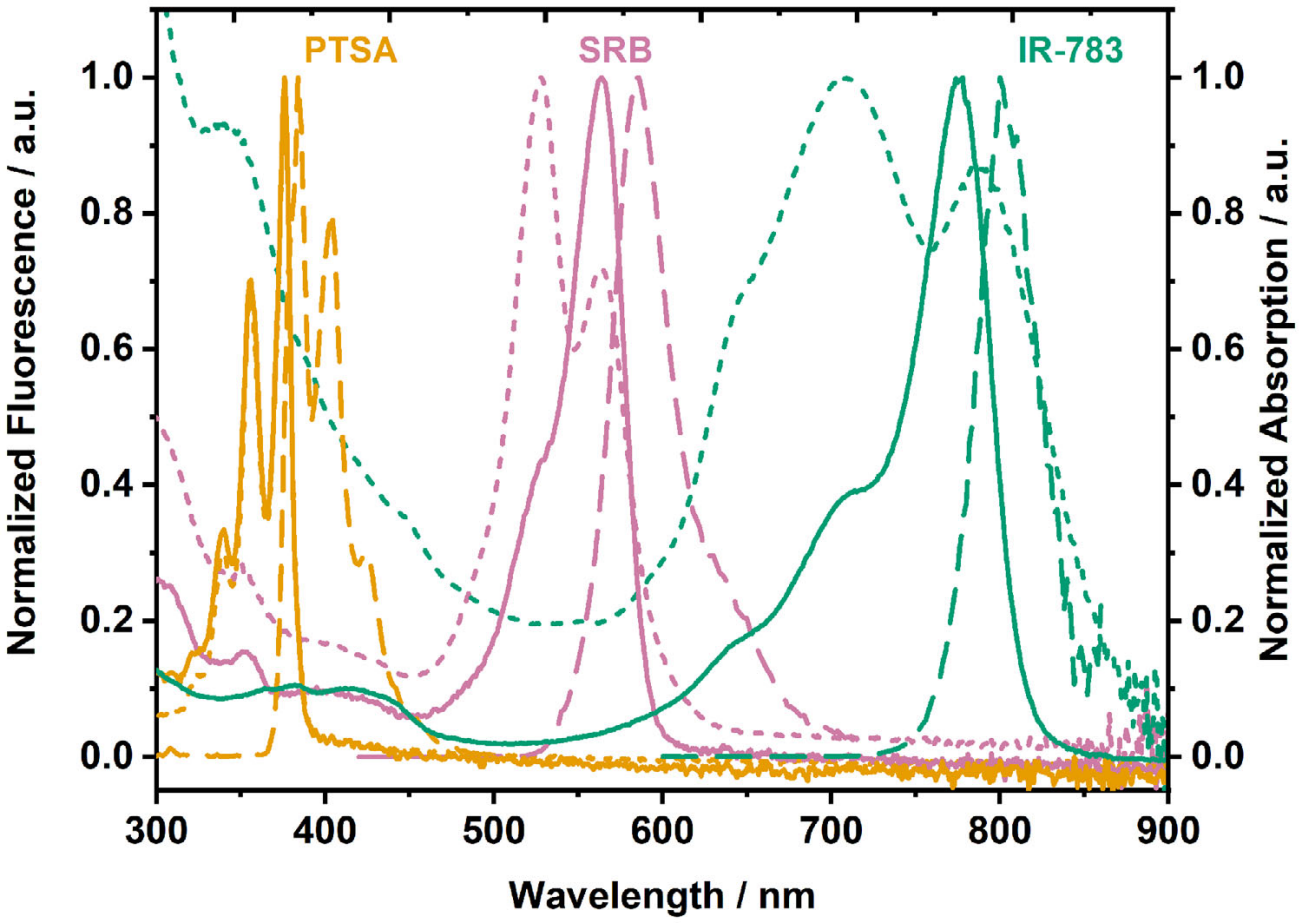
Optical spectra of SRB, PTSA, and IR‐783. Solid lines show absorbance spectra, dashed lines represent fluorescence spectra and dotted lines refer to dyes encapsulated in liposomes (44%mol. cholesterol lipid mixture, PTSA‐Liposome‐2, SRB‐Liposome‐4, IR‐Liposome‐2). 20 µm total lipid concentration of each dye encapsulating liposome was measured.

**Figure 2:**
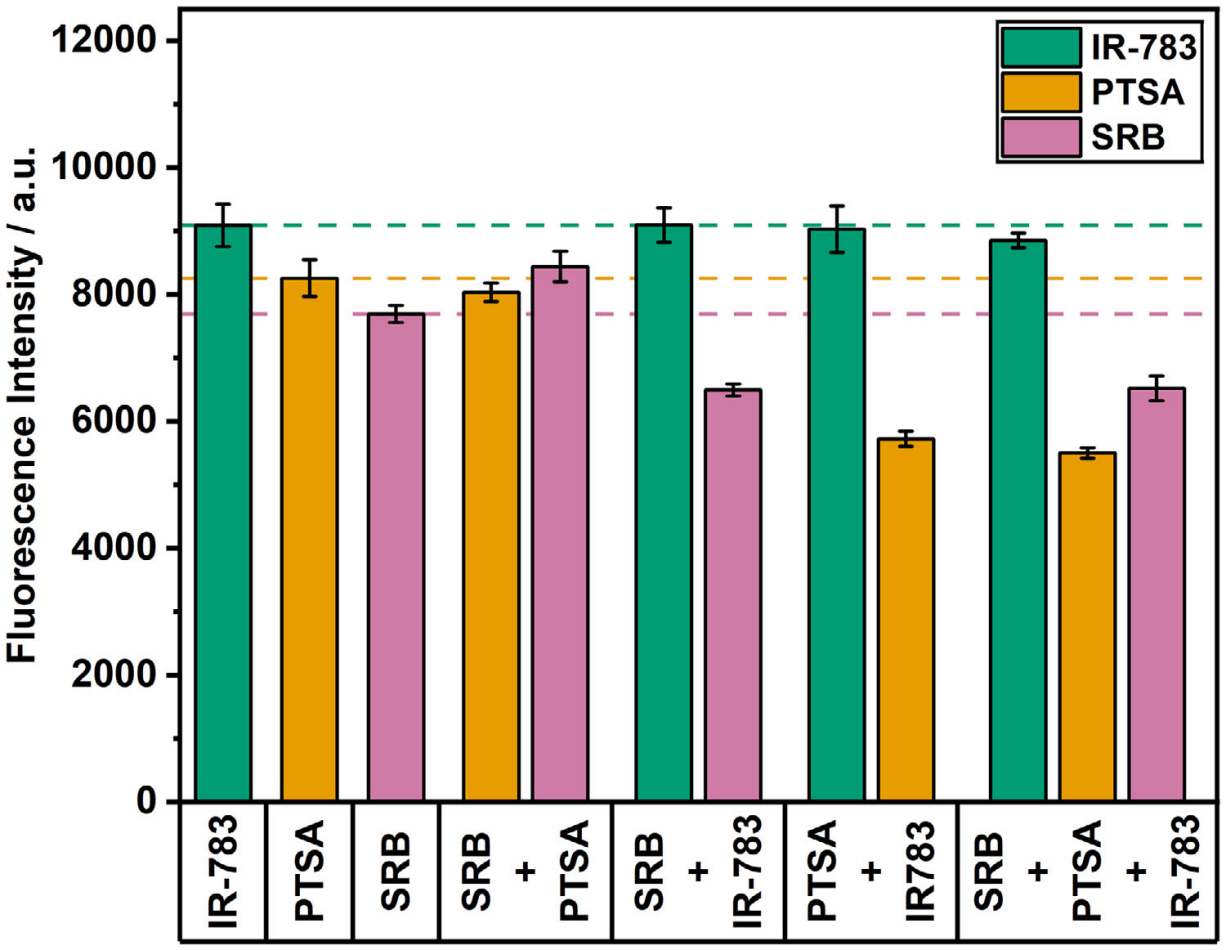
Fluorescence intensities of PTSA, SRB, and IR‐783 in different compositions (each fluorophore at 10 µm). Reference lines and control bars show the fluorescence intensities of pure dyes. IR‐783, λ_Ex_ = 790 (13) nm, λ_Em_ = 820 (13) nm, gain 150; SRB, λ_Ex_ = 565 (8) nm and λ_Em_ = 585 (8) nm, gain 75; PTSA, λ_Ex_ = 375 (5) nm and λ_Em_ = 405 (5) nm, gain 100; *n* = 3.

In bioassays, the simultaneous detection of several analytes in the same sample is often desirable since it allows more data per assay, increases efficiency, reduces reagent consumption, and shortens assay times. Thus, multiplex bioassays are often more cost‐effective in diagnostic setups. The selection of the three fluorophores used in this work, which are encapsulated into the liposomes, was aimed exactly at the development of such a multiplex detection system. The fluorophores were carefully selected to allow this simultaneous detection by showing minimal overlap in their optical spectra. Since liposomes encapsulating SRB have already been used for more than two decades^[^ ^24^ ^]^ with extensive analysis and optimization of encapsulant concentration and lipid composition,^[^ ^25^ ^]^ they were used as standard reference liposomes. Consequently, PTSA was chosen as a fluorophore in the lower wavelength range and IR‐783 in the higher wavelength range. Spectral overlap was avoided as much as possible (**Figure** **1**). In general, simultaneous detection of the free dyes could be confirmed (**Figure** **2**) and the minimal effects on the mutual emission can be corrected mathematically. Specifically, while the equimolar combination of PTSA and SRB only minimally influenced the fluorescence signal of each other, the presence of IR‐783 detectably reduced the fluorescence obtained for the other two dyes, albeit to a small degree. The mixture of PTSA and SRB caused an increase in SRB signal of 10 ± 4%, while the signal of PTSA showed an insignificant reduction of 3 ± 4% compared to the pure dyes. Thus, SRB partially reabsorbs PTSA emission, resulting in decreased PTSA and increased SRB intensities. The IR‐783 signal intensity was unchanged for all mixtures compared to the pure dye. The PTSA signal intensity dropped by 31 ± 3% when mixed with IR‐783 and by 33 ± 3% when mixed with both other dyes simultaneously. SRB fluorescence was quenched by 16 ± 2% when mixed with IR‐783 and by 15 ± 3% when mixed with the other two dyes. Hence, not surprisingly, IR‐783 influences the fluorescence intensities of the other two dyes due to its broad absorption bands, due to significant radiative energy transfer at the given concentrations, and possibly by heteromolecular aggregation effects between the fluorophore molecules. The latter could likely be caused by *π*–*π*‐interactions of the aromatic moieties found in all investigated fluorophores.

### 2.1 Liposome Preparation and Characterization

**Figure 3:**
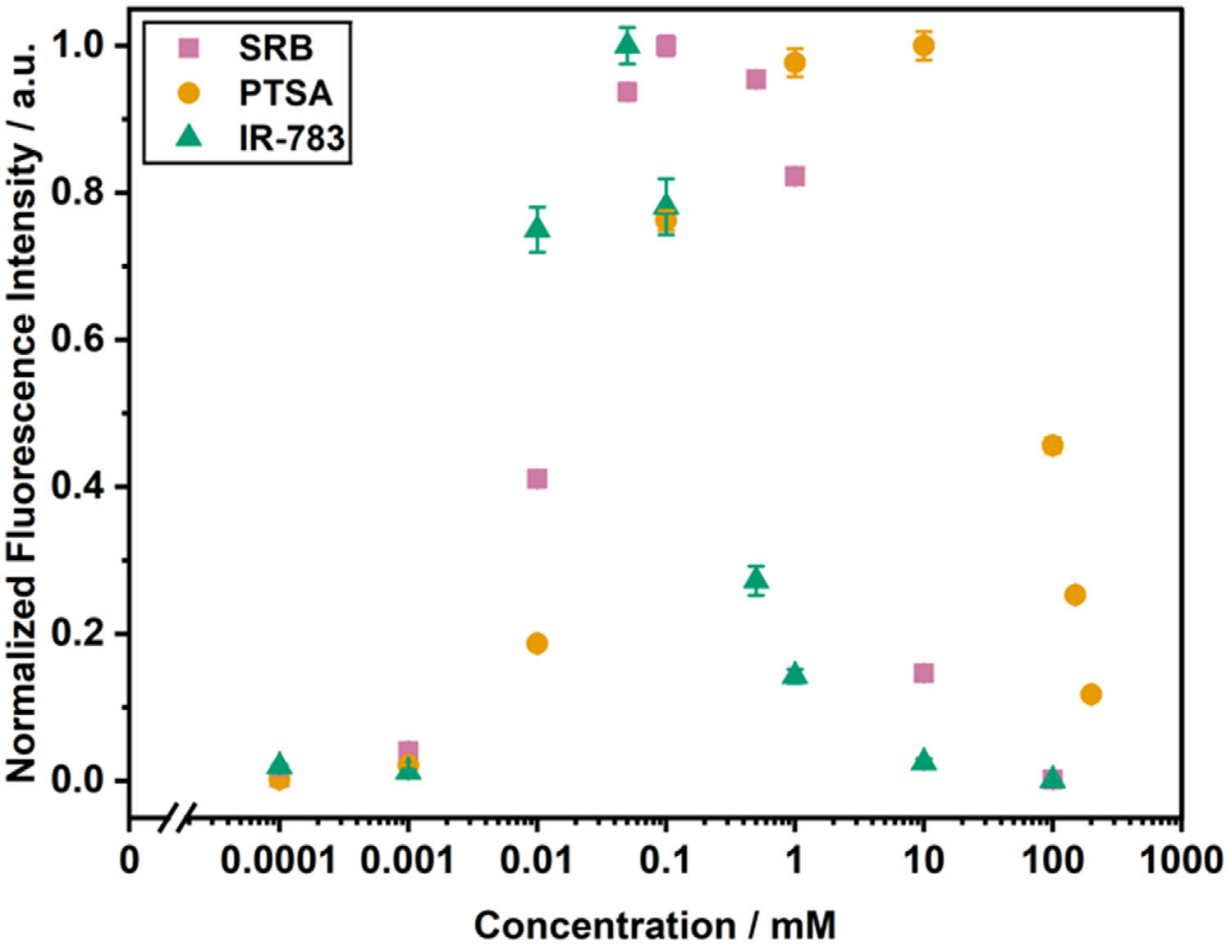
Normalized fluorescence intensities for fluorophore titrations in Liposome Complement Buffer (LCB). Normalization to maximum intensity. IR‐783, λ_Ex_ = 790 (13) nm, λ_Em_ = 820 (13) nm, gain 150; SRB, λ_Ex_ = 565 (8) nm and λ_Em_ = 585 (8) nm, gain 75; PTSA, λ_Ex_ = 375 (5) nm and λ_Em_ = 405 (5) nm, gain 100; n = 3 (except IR‐783 0.0001 mM and PTSA 0.01 mM, *n* = 2).

High concentrations of fluorescent dyes lead to aggregation. Depending on the relative orientation of the chromophores, H‐ or J‐type molecular aggregates can be formed.^[^ ^26^, ^27^ ^]^ H‐aggregates usually exhibit a blueshifted absorption and reduced emission compared to the monomeric dye. For both SRB and IR‐783, corresponding characteristics are found in the absorption spectrum of the dyes (dotted line in Figure 1). This is in line with the observation that intact liposomes exhibit no or minimal fluorescence (associated with the common term ‘self‐quenching’), whereas in their lysed state, when the encapsulants are released and diluted in the surrounding solution, high fluorescence signals are observed, allowing easy quantification of their release.^[^ ^28^ ^]^ For SRB, liposome dye concentrations ranging from 10 to 150 mM have been described to show this phenomenon,^[^ ^25^, ^29^, ^30^ ^]^ i.e. efficient self‐quenching (as explained above) could be confirmed in this concentration range (**Figure** **3**). Subsequently, an SRB encapsulant concentration of 10 mM was chosen, which resulted in a signal intensity of 15 ± 1% when encapsulated compared to the maximum signal upon fluorophore release. The fluorophore IR‐783 showed self‐quenching due to aggregation with the lowest concentrations among the investigated dyes starting at 0.05 and ≈1 mM aggregates are clearly predominant (Figure 3). Hence, similar to SRB, 10 mM IR‐783 was chosen with low background signals of only 2.5 ± 0.5% of the released maximum signal. For PTSA, higher concentrations were necessary to ensure efficient self‐quenching (Figure 3). Dye concentrations of 150 mM and above were determined to be suitable with residual signal intensities of 25.3 ± 0.5% for 150 mM and 11.7 ± 0.3% for 200 mM of the released maximum intensity. In previous work, preparation conditions for liposomes have been established for SRB liposomes with encapsulant concentrations of 10 mM SRB and 210 mM NaCl. Due to the structure of PTSA with its four sulfonic acid groups, concentrations above 100 mM PTSA bear the risk of a too high osmolality inside the liposomes compared to the osmolality of the established buffers for SRB liposome preparation. This can lead to liposome instability or fluorophore leakage if the buffers are not adjusted accordingly. Therefore, although fluorescence signal reduction was not ideal at 100 mM, the concentration range of 100, 150, and 200 mM PTSA was tested as an encapsulant. With this, self‐quenching in the final system could be investigated and it could be evaluated whether higher concentrations with potential buffer adjustments are mandatory. Ultimately, for multiplex analyses, all types of liposomes must be stable under the same buffer conditions, hence similar osmolalities are mandatory to avoid fluorophore leakage or liposome swelling.

Anionic liposomes were prepared by reverse‐phase evaporation following the established method by Edwards et al.^[^ ^31^ ^]^ Liposome preparation was examined with high (i.e. 44%mol.) and low (i.e. 5%mol.) cholesterol lipid mixtures in combination with each dye separately to investigate the effect of the respective dye on the different analytically typical lipid compositions.^[^ ^25^ ^]^ Liposomes prepared with 10 mM SRB and 210 mM NaCl as encapsulant and extruded through the smallest membrane pore size of 0.2 µm resulted in vesicles with a diameter of 143 ± 2 nm for high cholesterol and 130 ± 1 nm for low cholesterol liposomes with a PDI of 0.10 ± 0.01 and 0.12 ± 0.01, respectively. The surface zeta potential of ‐25 ± 1 mV for high cholesterol and −16 ± 1 mV for low cholesterol liposomes confirmed the expected anionic characteristics contributing to their colloidal stability. Purification by size exclusion chromatography (SEC) led to a clear separation of the liposome and the excess dye band. Consequently, dialysis as a second step to remove excess encapsulant could be kept short over one night including two buffer exchanges.

Similar results were obtained for PTSA liposomes. Preparation parameters were not changed except for the encapsulant containing either 100, 150, or 200 mM PTSA without additional sodium chloride. The sodium chloride and sucrose content in the respective liposome storage buffer was adjusted from 200 mM NaCl and 200 mM sucrose to 250 mM NaCl and 335 mM sucrose for 150 mM and 400 mM NaCl and 300 mM sucrose for 200 mM PTSA encapsulating liposomes. The resulting liposomes were slightly larger in diameter with 147 to 165 nm for the high cholesterol and in a similar size range with 118 to 142 nm for the low cholesterol lipid composition compared to SRB liposomes, with no trend regarding the encapsulated PTSA content. The determined PDI was in the range of 0.06 to 0.08 and 0.09 to 0.12, respectively. The surface zeta potential was in a comparable range to SRB liposomes with −19 to −16 mV for the low cholesterol and −22 to ‐18 mV for the high cholesterol liposome batch. As for SRB liposomes, preparations with PTSA showed a clear separation of liposome and free fluorophore band during SEC, which resulted in the same short dialysis time.

Liposome preparations with IR‐783 (i.e. 10 mM IR‐783 and 210 mM NaCl) as encapsulant resulted in liposome formation only for the high cholesterol lipid composition. For the low cholesterol preparation, the lack of a second band during SEC indicated an absence of liposomes. This observation was further substantiated through DLS measurements which confirmed the absence of vesicles (data not shown). For high cholesterol compositions, worse separation of excess dye and liposomes was observed during SEC, leading to the use of longer (i.e. ≈45 cm instead of ≈15 cm in length) and wider (i.e. diameter increased from 1.5 to 3.0 cm) columns. In addition, an extended dialysis time of two days compared to preparations with the other two dyes was required to remove all the excess encapsulant. The obtained liposomes extruded through the smallest pore size of 0.4 µm had a diameter of 170 ± 10 nm. The PDI of 0.26 ± 0.01 indicates a broader size distribution compared to the other liposomes, which can be attributed to the use of the larger membrane pore size of 0.4 µm instead of 0.2 µm as for the other liposomes. The measured surface zeta potential for IR‐783 containing liposomes of −23 ± 3 mV was in a similar range to liposomes with the other two dyes.

Overall, liposomes could be successfully synthesized with all dyes except the low‐cholesterol lipid compositions in combination with IR‐783 in the encapsulant. This finding gave rise to the assumption that each dye may have unique characteristics as an encapsulant that may also influence the lipid arrangement, preventing liposome formation in the case of IR‐783. The assumption was further supported by the increased effort needed to separate the excess dye from the liposomes.

### 2.2 Optical Characterization

Encapsulation of 10 mM SRB inside the liposomes resulted in an initial fluorescence of 6 to 12%, as determined by comparing the fluorescence signal intensity of intact and lysed liposomes by detergent addition. Even lower initial fluorescence values were achieved when IR‐783 was encapsulated. Here, values as low as 1% were obtained. The notable reduction of the fluorescence signal found for the dyes confined in intact liposomes points toward the formation of H‐aggregates. By increasing the amount of PTSA in the encapsulant solution, the initial fluorescence of high cholesterol liposomes was improved from 40 ± 2% (100 mM PTSA) to 25 ± 1% (150 mM PTSA) and 13 ± 1% (200 mM PTSA), resulting in an overall better resolution between lysed and non‐lysed liposome signals. Higher concentrations are required for pronounced self‐quenching effects with PTSA, as can be seen from Figure 1 where only slight changes in the absorption spectrum for the liposome sample is evident at the given concentration, in contrast to the situation for the other two dyes. However, osmolality‐induced lysis in the assay buffer was observed through increased fluorescence signals for 150 mM and 200 mM PTSA liposomes (Figure S1, Supporting Information). This was partially resolved by increasing the sugar content of the assay buffer from 200 to 440 mM for 150 mM PTSA liposomes or to 660 mM for 200 mM PTSA liposomes (Figure S1, Supporting Information). Conversely, high concentrations of sucrose and thus a high osmolality in the buffer surrounding the liposomes could interfere with the multiplex application combining all liposome types together. Hence, the medium concentration of 150 mM PTSA for the encapsulant was chosen as a compromise to test the interaction with biological systems, in this case the human complement system.

The previously mentioned observations during liposome preparation suggest that the dyes influence the lipid bilayer formation during preparation and thus interact directly with the bilayer. A very simple assay was devised to qualitatively demonstrate the differences between the three dyes. Specifically, liposomes without encapsulated dye (ghostisomes) were incubated with each dye from the outside in the surrounding buffer, followed by dialysis against pure buffer over 24 h. The assay was performed for both a high and a low‐cholesterol version of the liposomes.

**Figure 4:**
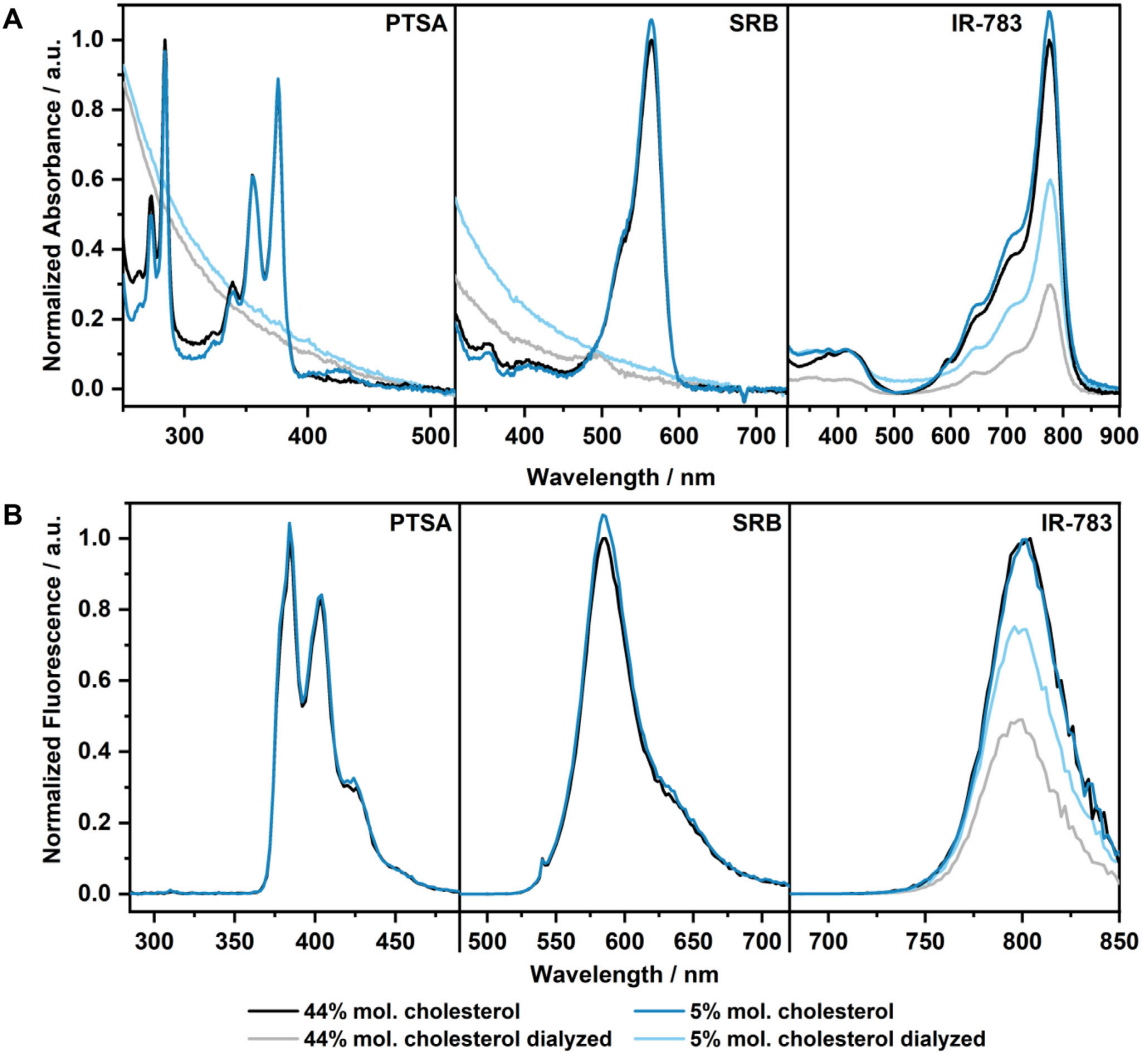
Absorption A) and emission spectra B) of dye‐ghostisome (Ghostisome‐1 and 3) incubation experiments with different cholesterol content with and without dialysis. Concentrations of PTSA and SRB were 2 µm without dialysis and 200 µm with dialysis. For IR‐783 concentrations of 10 µM were chosen for all samples. The excitation wavelengths for all IR‐783 samples were λ_Ex_ = 777 nm, for PTSA samples λ_Ex_ = 284 nm, and for SRB‐samples λ_Ex_ = 540 nm. For PTSA and SRB, no more characteristic dye absorption but rather only scattering signals (as identified by the λ^−4^ dependence) were observed for the dialyzed samples, which is why also no emission signals are plotted for them. Note that all curves were normalized with respect to the Ghostisome with 44%mol. Cholesterol (black lines).

Ghostisome preparations yielded liposomes with a diameter of 301 ± 7 nm for high cholesterol and 220 ± 11 nm for low cholesterol lipid compositions with a PDI of 0.16 ± 0.02 and 0.20 ± 0.01, respectively. The absorbance spectra measured after incubation with the dye and subsequent dialysis indicate that SRB and PTSA may interact with the membrane, however, they can be completely dialyzed away from the liposomes (**Figure** **4**). In fact, samples after dialysis were measured 100 times more concentrated than the control sample, and still no characteristic absorbance was detected at the respective wavelengths which would be indicative for residual dye, but only a scattering background was observed. This leads to the inference that no or only weak and reversible interactions with the lipid membrane are present for both dyes.

In contrast, the fluorophore IR‐783 could not be completely removed by dialysis over 24 h (Figure 4). Significant absorbance remained in the dialyzed samples of both liposome versions and fluorescence could be detected (Figure 4B), even given the low quantum yield and short fluorescence lifetime of this dye,^[^ ^32^ ^]^ indicating that a significant amount of it is still present in the dialyzed samples. For the low cholesterol liposomes, the absorbance was reduced by 47% compared to the non‐dialyzed control sample (calculated at the absorbance maximum at 777 nm). For the high cholesterol liposomes, dialysis reduced the absorbance by 66% (calculated at the absorbance maximum at 777 nm). From this, it can be concluded that the fluorophore IR‐783 interacts with the lipid bilayer in a non‐reversible manner which resists removal by dialysis. Furthermore, the greater decrease in absorbance for the high‐cholesterol liposomes suggests a weaker interaction of the fluorophore IR‐783 with this lipid composition compared to the low‐cholesterol liposomes. This finding is consistent with the observation that the preparation of low‐cholesterol liposomes in combination with IR‐783 was not achieved, whereas the preparation of high‐cholesterol liposomes was successful. This may be attributed to too excessive interaction of IR‐783 with the lipids, which may prevent successful liposome formation.

### 2.3 Lipid Bilayer Characterization by SAXS

**Figure 5:**
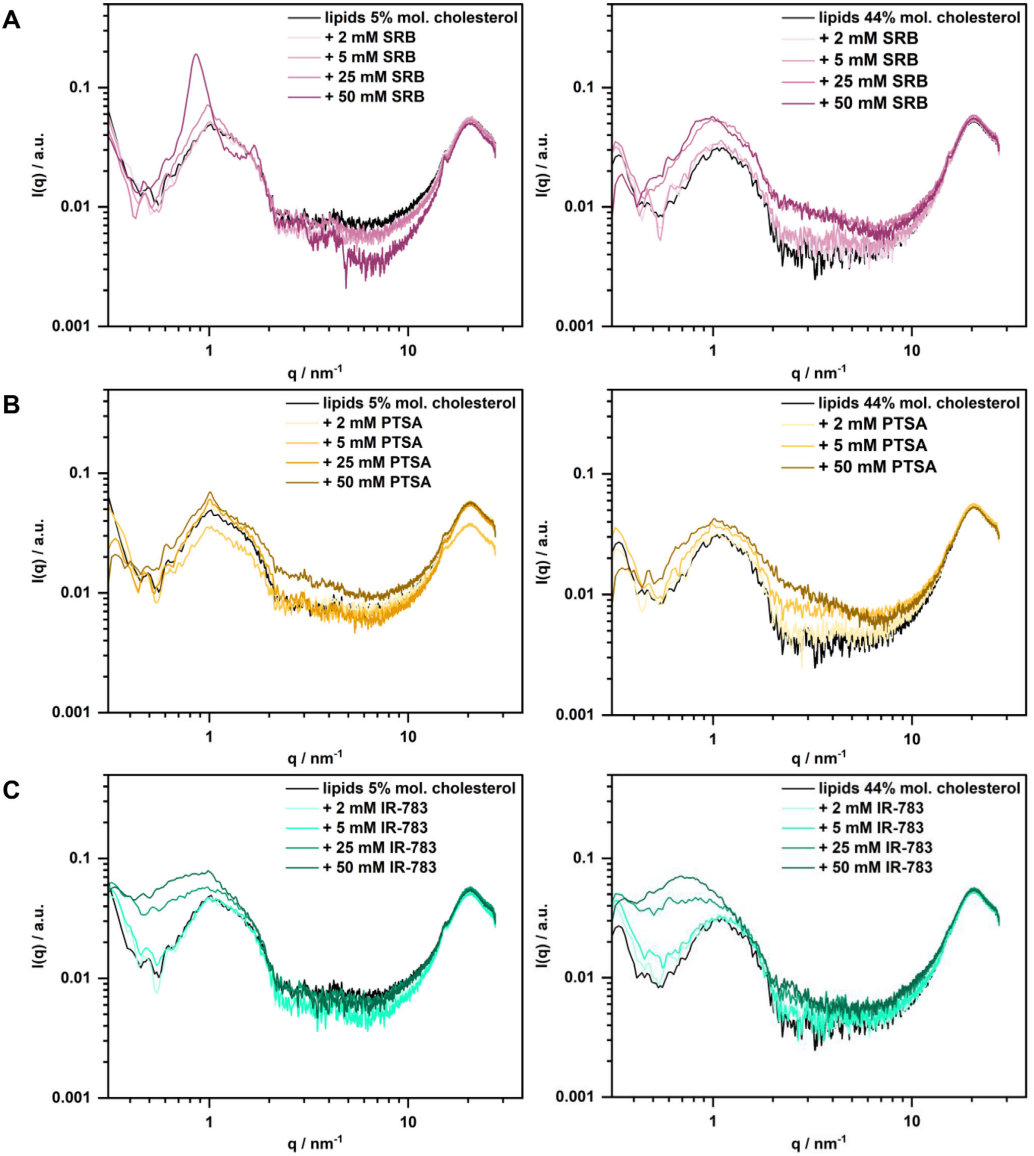
SAXS measurements with lipid mixtures (50 mM total lipid concentration, left: 5%mol. cholesterol lipid mixture, right: 44%mol. cholesterol lipid mixture) and fluorophore titration from 2 to 50 mM (A: SRB, B: PTSA, C: IR‐783).

Further insight into the dye‐lipid bilayer interactions was obtained through SAXS measurements. Specifically, each dye was mixed and investigated at different concentrations ranging from 2 to 50 mM with high and low‐cholesterol lipid mixtures, maintaining a constant total lipid concentration of 50 mM. The pure lipid samples analyzed as controls showed a form factor typical for lipid bilayer, showing a large oscillation between 0.5 and 2.2 nm^−1^ with a local minimum at 0.5 nm^−1^, and a slightly pronounced Bragg peak around q  =  1.06 nm^−1^ (**Figure** **5**), caused by smectic ordering.^[^ ^17^, ^33^ ^]^ This peak indicates an interlamellar distance of 5.93 nm, i.e. the lamellar repeat distance in stacked bilayers, which is in accordance with values reported in the literature.^[^ ^34^, ^35^ ^]^ The form factor did not change when SRB was added to the lipid mixtures, regardless of whether the cholesterol content was high or low (Figure 5A). However, for the low cholesterol compositions, the intensity of the peak (q = 1.06 nm^−1^) increased with high SRB concentrations (≥ 25 mM), which can be attributed to a higher order in the multilayer system created by stacking additional bilayers. Furthermore, the peak shifted to a lower q value of 0.88 nm^−1^ at an SRB concentration of 50 mM, indicating that the distance between the bilayers increased from 5.92 to 7.13 nm. We hypothesize that this swelling of the multilayers is due to SRB adsorption on the bilayer surface, causing increased electrostatic repulsion between bilayers, as usually observed upon electrostatically charging bilayers.^[^ ^36^ ^]^ SRB is indeed a charged species, which therefore charges the bilayer when it adsorbs on it. Noteworthy is that the overall bilayer structure (form factor) remains unchanged upon the addition of SRB, informing that SRB adsorbs onto the bilayer surface without significantly altering its electron density profile (bilayer structure). It is likely that the surface concentration of SRB at the bilayer surface is too low to affect significantly the overall electron density at the top bilayer surface.

When PTSA was added to the lipid solutions, results similar to those of SRB lipid mixtures were obtained, with the overall form factor remaining unchanged (Figure 5B). Especially for the high cholesterol‐containing sample, no influence of PTSA on the bilayer was found. Lipid mixtures with low cholesterol content partially showed a peak emerging at ≈1 nm^−1^, which is comparable to the one observed with SRB at the same concentration but much less pronounced. However, here no shift in the peak position was observed upon the addition of PTSA. Hence, we can conclude that PTSA does not interact with the lipid membrane, neither by adsorption nor by altering the lipid arrangement in the bilayer.

In contrast, the presence of the fluorophore IR‐783 in lipid mixtures had a significant effect on the bilayer form factor in the q‐range from 0.3 to 1 changed by increasing IR‐783 concentration from 5 to 50 mM (Figure 5C). For both high and low‐cholesterol lipid mixtures, a distinct shift in the position of the local scattered intensity minimum of the bilayer form factor from 0.5 down to 0.4 nm^−1^ upon addition of IR‐783 and an excess scattering in the region of the large oscillation are observed. This observation indicates that the fluorophore strongly affects the bilayer structure, either by locating in the bilayer surface, i.e. in the polar head region, and/or in the hydrophobic core of the lipid bilayer, thereby substantially altering the electron density profile that produces the pattern probed by SAXS (bilayer form factor). Interestingly, IR‐783 has been shown to be membrane‐permeable through transport mechanisms only. Specifically, IR‐783 enters cells primarily through organic anion transporter polypeptides (OATPs), which are overexpressed in many cancer cells. This mechanism allows IR‐783 to selectively accumulate in tumor cells, making it useful for imaging and therapeutic applications.^[^ ^37^, ^38^ ^]^ Once inside the cells, it localizes to the mitochondria, where it is described to induce mitochondrial fission and to affect cellular processes such as apoptosis and ATP production.^[^ ^39^, ^40^ ^]^ Based on the data presented here, it needs to be considered that IR‐783 may not only enter cells via OATPs but also through the hydrophobic areas of the lipid bilayers.

Subsequently, these results were confirmed for liposomes encapsulating the respective fluorophores. To generate sufficiently high signal intensities, synthesized fluorophore encapsulating 5 mM liposome stock solutions were concentrated ≈10‐fold by water evaporation. Again, a significant change in the form factor of the bilayer was obtained only for liposomes encapsulating IR‐783 (Figure S2, Supporting Information). Differences in signal intensity between the different liposome samples are probably due to slight variations in the lipid concentration of the samples since the concentration was estimated by eye during the preconcentration step based on the residual volume in the reaction tubes.

### 2.4 Reactivity Toward Biological Matrices

The interaction of encapsulants with the lipid bilayer is of relevance in any drug delivery or bioanalytical assay, since the liposome surface chemistry is typically carefully engineered for each specific application. Therefore, to investigate the potential impact these changes in bilayer structure and the interaction of the lipid surfaces with the fluorophores may have, a biological interaction assay was performed which assesses liposome surface interactions with the ever‐present complement proteins in human serum. Liposomes can be designed to avoid recognition by the complement system and to remain stable in human serum. However, changes in surface chemistry and the use of ligands can cause the activation of the complement system, leading to opsonization and ultimately liposome lysis.^[^ ^25^ ^]^ Here, fluorophore deposition or alterations in the lipid membrane are expected to influence the reactivity of the complement system with these liposomes.

**Figure 6:**
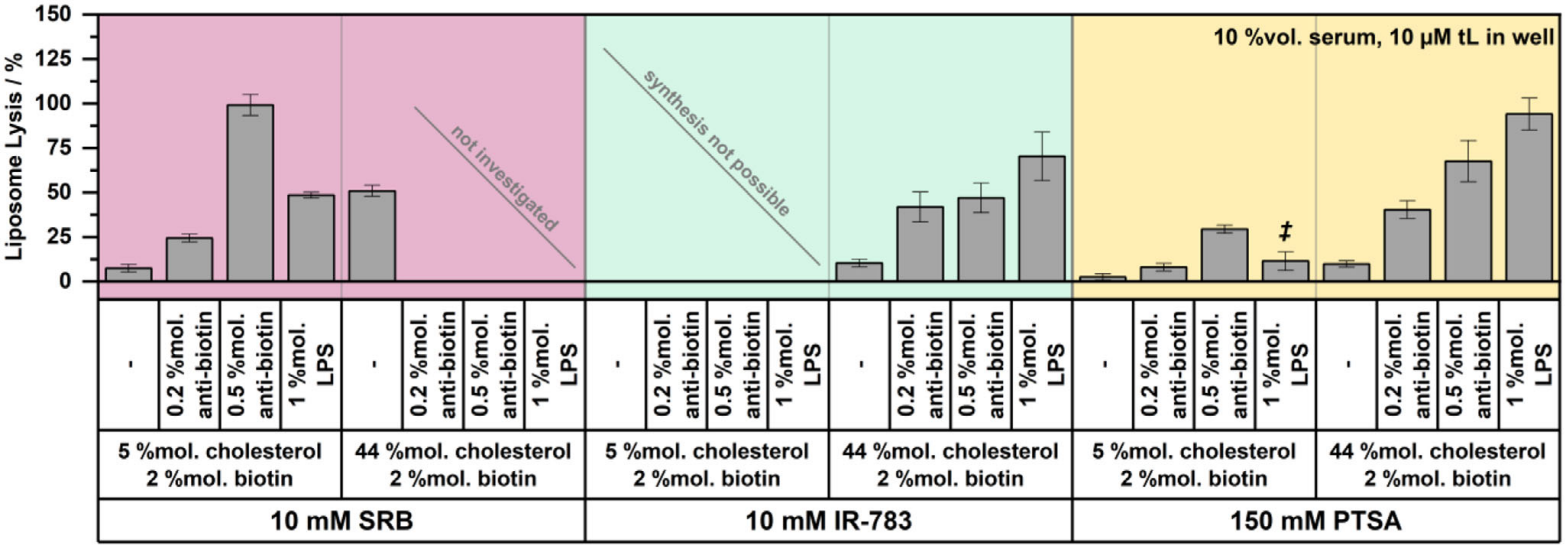
Liposome‐based complement assays determining the reactivity of the human complement system toward varying liposome surfaces and encapsulants (SRB, IR‐783, or PTSA). Liposomes with low and high cholesterol content with and without surface modifications (SRB‐Liposome‐1, 2 , and 3, IR‐Liposome‐1 and 3, PTSA‐Liposome‐3, 4, 5 , and 6). Antibodies were incubated for 15 min at RT with the liposomes prior to the complement assay. Lipopolysaccharides were included into the lipid composition during liposome preparation. Fluorescence intensities were corrected by inactive serum controls as background and normalized to a detergent containing positive control (30 mM OG). Liposomes were measured at 10 µm tL. Complement assays were performed in LCB with 200 mM sucrose for SRB and IR‐783 liposomes and 440 mM sucrose for PTSA liposomes. Serum content for all liposomes was 10 %vol. per well. *‡* low cholesterol PTSA LPS liposomes were measured with a different serum batch. *n* = 3.

Such a bioassay was developed and optimized for 10 mM SRB encapsulating liposomes. While high cholesterol contents are known to activate the complement system,^[^ ^41^, ^42^, ^43^ ^]^ a reduction of the cholesterol content results in stable liposomes in serum over the investigated period of 1 h at 37 °C.^[^ ^25^ ^]^ In addition, surface‐bound antibodies in sufficient density on the surface are well known to be complement triggers^[^ ^44^ ^]^ and are also able to induce a dose‐dependent complement activation with low cholesterol SRB liposomes (0.2%mol. antibody: 24 ± 2% lysis, 0.5%mol. antibody: 99 ± 5% lysis, **Figure** **6**). Furthermore, lipopolysaccharides (LPS) from bacterial membranes can cause both antibody‐mediated and antibody‐independent complement activation.^[^ ^45^, ^46^ ^]^ For this reason, experiments were designed here to study the effect of plain liposomes, LPS‐tagged liposomes, and antibody‐bound liposomes (Figure 6). As expected, all low‐cholesterol liposomes encapsulating SRB or PTSA that did not contain a complement trigger were not lysed, whereas high‐cholesterol SRB liposomes were lysed. Surprisingly, high cholesterol liposomes entrapping PTSA or IR‐783 were stable. This suggests that not cholesterol alone, but in conjunction with SRB, which has been shown to be adsorbed on the bilayer structure by the SAXS analyses, acts as a complement trigger. Consequently, experiments using antibody or LPS triggers exhibited similar responses for the high cholesterol PTSA and IR‐783 and the low cholesterol SRB liposomes. Low cholesterol PTSA LPS liposomes were not stable over time and showed fluorophore leakage (data not shown). Therefore, they were only investigated in the complement assay right after preparation with a different serum batch. Nevertheless, the trend of significantly lower responses by the low cholesterol PTSA liposomes, with low antibody concentrations and LPS only being able to activate the complement system very weakly, is still visible and in contrast to low cholesterol SRB liposomes. It can be assumed that the high cholesterol content may be needed to assist the complement response. This may be due to a better surface for complement protein deposition by cholesterol, hence free hydroxy group cluster formation.^[^ ^47^, ^48^ ^]^ In addition, the cholesterol content also influences membrane characteristics such as membrane fluidity and thickness,^[^ ^49^, ^50^ ^]^ which effect this has on complement‐mediated liposome lysis is still unclear.

These findings are important in clarifying statements made in literature regarding the activation of the complement system by cholesterol and in assigning it a more assisting role than a triggering role by itself. More broadly seen, however, these studies emphasize the relevance of gaining an understanding of the interactions encapsulants have with the chosen lipid bilayer, as they will clearly impact the often carefully designed liposome surface chemistry and lead to unexpected interactions with matrix components.

## 3 Conclusion

In summary, the present study demonstrates that it is indeed very relevant to investigate the effects entrapped molecules may have on liposome characteristics and the reactivity of biological systems toward these liposomes. This was demonstrated by examining fluorophores as model liposome loadings, which, besides inducing variability in the optical characteristics, can significantly influence the lipid bilayer membrane characteristics of the liposomes. Consequently, the reactivity of the final liposomes toward biological matrices and also the overall success of liposome preparations is dependent on the encapsulated molecule, in this case the fluorophore. While fluorophore incubation experiments and SAXS measurements confirmed alterations in the lipid arrangements for IR‐783, the liposome‐based complement assays revealed that lipid compositions or surface modifications cannot be simply transferred between various types of liposomes encapsulating different fluorophores. This generally emphasizes that the encapsulant itself, which initially may seem to have no impact on the outer surface characteristics, indeed plays a crucial role in the biological stability of liposomes. Although the primary focus in assessing liposome stability in biological environments is often limited to the examination of lipid composition, surface modification, and general parameters such as temperature, pH, liposome size, surface charge, or osmolality, a comprehensive evaluation of both surface chemistry and the influence of the encapsulant is essential for the optimization of liposomes tailored to their targeted applications. This aspect is of particular relevance in areas such as drug delivery or diagnostic imaging, where long blood circulation times are critical for liposomal drug efficacy and contrast agent efficiency.

## Conflict of Interest

The authors declare no conflict of interest.

## Supporting information



Supporting Information

## Data Availability

The data that support the findings of this study are available from the corresponding author upon reasonable request.
