# Supplementary material for: Encapsulants Affect Liposome Surface Interactions with Biological Systems
Source: Small. 2025 Jun 19;21(33):2505312. doi: 10.1002/smll.202505312 (PMC12372433; doi:10.1002/smll.202505312)
Supplement: Supplementary file 1 — Supporting Information [file SMLL-21-2505312-s001.pdf]

NANO · MICRO
small

Supporting Information

for *Small*, DOI 10.1002/smll.202505312

Encapsulants Affect Liposome Surface Interactions with Biological Systems

*Clemens Spitzenberg, Christoph Bruckschlegel, Ferdinand Holzhausen, Sebastian Boesl-Bichlmeier, Coralie Pasquier, Patrick Nuernberger, Pierre Bauduin and Antje J. Baeumner**

Supporting Information

Encapsulants Affect Liposome Surface Interactions with Biological Systems

Clemens Spitzenberg[a],[+], Christoph Bruckschlegel[a],[+], Ferdinand Holzhausen[a],[+], Sebastian Boesl-Bichlmeier[b], Coralie Pasquier[c], Patrick Nuernberger[b], Pierre Bauduin[c], Antje J. Baeumner[a],*

[a] M.Sc. C. Spitzenberg, Dr. C. Bruckschlegel, M.Sc. F. Holzhausen, Prof. Dr. A. J. Baeumner
Institut für Analytische Chemie, Chemo- und Biosensorik,
Universität Regensburg
D-93053 Regensburg, Germany
E-mail: antje.baeumner@ur.de

[b] M.Sc. S. Boesl-Bichlmeier, Prof. Dr. P. Nuernberger
Institut für Physikalische und Theoretische Chemie,
Universität Regensburg
D-93053 Regensburg, Germany

[c] Dr. C. Pasquier, Dr. P. Bauduin
ICSM, CEA, CNRS, ENSCM
University Montpellier
F-30207 Bagnols sur Cèze, France

[+] These authors contributed equally to this work.

WILEY-VCH

Experimental section

Chemicals and consumables

All chemicals were of analytical grade and used without purification. Sephadex® G-50 (G50150), Lipopolysaccharides (LPS) from Salmonella enteritidis (L6011), Cholesterol from sheep wool (C8667) and tetrasodium ethylenediaminetetraacetic acid (EDTA, EDS), ethylene glycol-bis(2-aminoethyl ether)-*N,N,N′,N′*-tetraacetic acid (EGTA, E4378), sodium hydroxide, methanol and polyclonal goat anti-biotin IgG (B3640) were purchased from Sigma-Aldrich/Merck KGaA (Darmstadt, Germany). Sodium azide and magnesium dichloride hexahydrate were purchased from Merck KGaA (Darmstadt, Germany). The extrusion kit and the phospholipids 1,2-dipalmitoyl-*sn*-glycero-3-phosphocholine (DPPC, 850355P), 1,2-dipalmitoyl-*sn*-glycero-3-phospho-(1′-*rac*-glycerol) (sodium salt) (DPPG, 840455P) and 1,2-dipalmitoyl-*sn*-glycero-3-phosphoethanolamine-*N*-(biotinyl) (DPPE-biotin, 870285P) were purchased from Avanti Polar Lipids (Alabaster, AL, USA). Sulforhodamine B (SRB, S1307) was purchased from Thermo Fisher Scientific (Darmstadt, Germany). Chloroform and nitric acid (HNO_3) were bought from Fisher Chemical (Schwerte, Germany). N-2-hydroxyethylpiperazine-N'-2-ethanesulfonic acid (HEPES, HN78.3), Sodium chloride, sucrose (4621.3), N-octyl-β-d-glucopyranoside (OG, CN23.3) and calcium chloride was bought from Carl Roth (Karlsruhe, Germany). Dialysis membrane Spectra/Por® 4 (MWCO 12 - 14 kDa, 132700) from Spectrum Laboratories and polycarbonate Whatman Nucleopore™ Track-Etched membranes with a pore size of 1.0 µm, 0.4 µm or 0.2 µm (Ø 19 mm) from Whatman were bought from VWR (Darmstadt, Germany). The ICP-OES phosphorus standard solution (10882.0000) was purchased from Bernd Kraft/AnalytiChem GmbH (Duisburg, Germany). Black 96-well flat bottom microtiter plates (MTP, 781608) were purchased from Brand (Wertheim, Germany). Pooled human complement serum was purchased from Innovative Research (ISCER, Novi, Michigan, USA). IR-783 (I1031) was purchased from TCI Deutschland GmbH (Eschborn, Germany). 1,3,6,8-pyrenetetrasulfonic acid (PTSA, P0021) was purchased from Chemodex AG (St. Gallen, Switzerland).

Liposome synthesis

Liposome synthesis was performed as described earlier[25] using the reverse-phase evaporation method. Standard anionic lipid composition contained 5 %mol. Cholesterol, 73 %mol. DPPC, 20 %mol. DPPG and 2 %mol. DPPE-Biotin for low cholesterol liposomes or 44 %mol. Cholesterol, 34 %mol. DPPC, 20 %mol. DPPG and 2 %mol. DPPE-Biotin for high cholesterol liposomes. For LPS liposomes, LPS from Salmonella enteritidis was added to the lipid mixture. For LPS an estimated molecular weight of 15000 $g \cdot mol^{-1}$ was used for calculations.
60 µmol of the desired lipids were dissolved in chloroform (3 mL) and methanol (0.5 mL). After sonication at 60 °C for 1 min, 2 mL of the preheated (60 °C) encapsulant solution was added. Encapsulant solutions contained 10 mM SRB, 210 mM NaCl or 10 mM IR-783, 210 mM NaCl or 100, 150 or 200 mM PTSA or 280 mM NaCl dissolved in 20 mM HEPES, pH 7.5. Mixture was sonicated again at 60 °C for 4 min. The organic solvents were removed with a rotary evaporator (LABOROTA 4001, Heidolph, Germany) at 60 °C while the pressure was reduced stepwise from 900 to 780 mbar within 40 min (900 mbar for 10 min, 850 mbar for 5 min, 800 mbar for 5 min, 780 mbar for 20 min). The solution was vortexed for 30 s, and another 2 mL of the encapsulant was added. After a second vortexing step for 30 s, the solution was further rotated at 60 °C while the pressure was reduced stepwise starting from 750 to 400 mbar within 60 min (750 mbar for 20 min, 600 mbar for 5 min, 500 mbar for 5 min, 400 mbar for 20 min). The solution was then extruded through polycarbonate membranes with varying membrane pore sizes (1 µm, 0.4 µm or 0.2 µm). Extrusion was done at 65 °C by pushing the solution with the syringes 21 times through each membrane pore size. Excess of encapsulant (i.e. fluorophore and NaCl) was removed by size exclusion chromatography with a Sephadex G-50 column followed by dialysis against the liposome storage buffer (HSS, 10 mM HEPES, 200 mM NaCl, 200 mM sucrose, 0.01 % NaN_3, pH 7.5). Liposomes were stored at 4 °C.
For PTSA encapsulating liposomes with a PTSA content of 150 and 200 mM PTSA the NaCl and sucrose content in the HSS buffer was adjusted to 250 mM NaCl, 335 mM sucrose and 400 mM NaCl, 300 mM sucrose respectively. Smallest membrane pore size during extrusion is mentioned in Table S1 for each liposome synthesis.

Liposome characterization

Dynamic light scattering (DLS), zeta potential and polydispersity index (PDI) measurements were performed on a Malvern Panalytical Zetasizer Nano-ZS. For size determination polymethyl methacrylate semi-micro cuvettes (Brand, Germany) and for zeta potential determination disposable folded capillary cells (Malvern Panalytical, Germany) were used. Measurement temperature was set to 25 °C with a 1:100 sample dilution in HSS buffer in both cases. Measurement of size and PDI were conducted with a dispersant refractive index n_D^{20} of 1.34, a material absorbance of zero, a dispersant viscosity η of 1.1185 mPa s, an angle of 173° and backscattering mode after equilibration for 15 s in three measurement runs with each 13 single measurements. For zeta potential, a dielectric

constant ε of 78.5 was used with an equilibration time of 60 s before four measurement runs started with each twenty single measurements.
Phospholipid concentrations were determined via optical emission spectroscopy with inductively coupled plasma (ICP-OES, SpectroBlue TI/EOP) from SPECTRO Analytical Instruments GmbH (Kleve, Germany). Liposomes solutions were diluted 1/150 with a total volume of 3 mL in 0.5 M HNO_3. A phosphorus standard diluted in 0.5 M HNO_3 from 1 to 100 µM was used for calibration of the device. Phosphorous was detected at a wavelength of 177.495 nm. Before each measurement, 0 µM and 100 µM phosphorus standard dilutions were used to re-calibrate the device. The total lipid concentration (tL) was calculated from the phospholipid concentration and the lipid composition used during synthesis.
The initial fluorescence of the liposomes was determined using a SYNERGY neo2 multi-mode reader from BioTek (Bad Friedrichshall, Germany). The liposome stock solutions were diluted to 50 µM tL concentration in HSS with and without 30 mM OG. Samples were prepared in quadruplicates. The so-called initial fluorescence was calculated as the ratio of the fluorescence intensities of intact (without OG) and lysed liposomes (with OG). Measurement conditions were λ_{Ex} = 565 (5) nm, λ_{Em} = 585 (5) nm, gain 100 for SRB liposomes, λ_{Ex} = 790 (13) nm, λ_{Em} = 820 (13) nm, gain 150 for IR-783 liposomes, and λ_{Ex} = 375 (5) nm, λ_{Em} = 405 (5) nm, gain 100 for PTSA liposomes.

Fluorophore mixtures and self-quenching

Fluorophores were dissolved in HSS to 100 mM in case of SRB and IR-783 and 200 mM for PTSA. Fluorophore mixtures were prepared out of these stocks with 10 µM of each respective dye. For the fluorophore self-quenching studies each fluorophore was investigated with a concentration titration from 1 µM to 100 mM for SRB and IR-783 and 1 µM to 200 mM for PTSA. Samples prepared in triplicates were measured with a SYNERGY neo2 multi-mode reader from BioTek (Bad Friedrichshall, Germany). Measurement settings: IR-783, λ_{Ex} = 790 (13) nm, λ_{Em} = 820 (13) nm, gain 150; SRB, λ_{Ex} = 565 (8) nm and λ_{Em} = 585 (8) nm, gain 75; PTSA, λ_{Ex} = 375 (5) nm and λ_{Em} = 405 (5) nm, gain 100.

Dialysis experiment

Ghostisome stock solutions were mixed with 100 mM dye stock solutions to reach 6 mM tL and 10 mM dye content in HSS and incubated for 1 hour at room temperature. From each sample a positive control without dialysis was taken. The remaining solutions were dialysed in parallel against pure buffer over 24 hours with two buffer exchanges. Dialysis was stopped at the same time for all samples. Total lipid concentration was determined again for all dialysed samples with the above described procedure.
For spectral investigations the solutions for PTSA and SRB were diluted to 2 µM. For the dialyzed samples a concentration of 200 µM was used. All solutions containing IR-783 were diluted to a concentration of 10 µM. All measurements were carried out with a cuvette with thickness 1 cm. Absorption spectra were recorded with the Cary 60 from Agilent. All spectra were referenced to the HSS buffer solution. The fluorescence measurements were carried out with the fluorimeter Fluorolog-3 from Horiba.

SAXS measurements

For SAXS measurements with lipid-dye mixtures high cholesterol (44 %mol. Cholesterol, 34 %mol. DPPC, 20 %mol. DPPG and 2 %mol. DPPE-Biotin) and low cholesterol (5 %mol. Cholesterol, 73 %mol. DPPC, 20 %mol. DPPG and 2 %mol. DPPE-Biotin) lipid stock solutions were dissolved to 100 mM tL in 20 mM HEPES, pH 7.5 at elevated temperatures while vortexing until a homogeneous mixture was obtained. 100 mM dye stock solutions were prepared in 20 mM HEPES, pH 7.5. Buffer, dye and lipid stock solutions were mixed accordingly to reach 50 mM tL with a varying dye content of 0, 2, 5, 25 or 50 mM.
For SAXS measurements investigating concentrated liposome solutions the respective liposomes were diluted to 5 mM in HSS followed by concentration through evaporation of water at room temperature with a rotary vacuum concentrator (RVC 2-18 CDplus HCI, Martin Christ Gefriertrocknungsanlagen GmbH, Germany). The process was stopped after reducing the volume by approximately 10 times.
SAXS measurements were performed on a bench built by XENOCS. The scattered beam of Mo radiation (λ= 0.071 nm) was collected on a 2D online scanner detector from MAR Research (diameter: 345 mm). Quartz capillaries (diameter: 2 mm) were used as sample containers and silver behenate was used for scattering vector calibration. Data treatment of raw data (integration) was conducted by pySAXS. Furthermore, the exact size of the capillaries was considered and background (empty capillary) as well as aqueous solvent (φ(water)= 50-70%) were subtracted from the liposome/lipid samples. All measurements were conducted at ambient temperature.

Homogeneous liposome-based complement assay

The liposome-based complement assay was performed following a previously established protocol.[25]
Each assay contained four liposome containing samples: liposomes in liposome complement buffer (LCB, 10 mM HEPES, 150 mM NaCl, 135 nM $CaCl_2$, and 1 mM $MgCl_2$ in double distilled H_2O, pH 7.4 prepared according to the HBS buffer of Zelek et al.[51], negative control), liposomes in active serum or in inactivated serum (negative control) containing 1/10 diluted inactivation buffer (iaCB, 200 mM EDTA and 0.5 µM EGTA, diluted in LCB, pH 7.5 – 8) and lysed liposomes by detergent addition as positive control, all prepared in LCB. Serum was inactivated by EDTA and EGTA addition to complex free Ca^{2+} and Mg^{2+} ions essential for the functionality of the complement system. Detergent positive controls contained 30 mM OG and serum in the final well. The final wells contained 100 µL volume, 10 µM tL liposomes and 200 mM sucrose for liposome stability. Final inactivated serum wells contained 20 mM EDTA and 0.05 µM EGTA. Serum content was 10 %vol. in the respective wells. All samples were prepared in triplicates.
Liposomes were prediluted in LCB to 100 µM tL solutions. In case antibodies were used as complement triggers, polyclonal goat anti-biotin antibodies (1.0 mg mL^{-1}) were incubated 15 min at RT with the liposome stock solution prior to dilution to 100 µM tL with LCB. Black 96 well, flat bottom microtiter plates were prepared on ice with LCB, sucrose (1 M sucrose in LCB), iaCB and OG (300 mM OG in double distilled H_2O) stock solution additions to the respective wells to reach the desired conditions. Liposomes were added, resulting in a 1/10 dilution in the final well. As last step, serum was added before incubation at 37 °C for 60 min and fluorescence intensity measurements with a SYNERGY neo2 multi-mode reader from BioTek (Bad Friedrichshall, Germany) (SRB: λ_{Ex} = 565 nm and λ_{Em} = 585 nm with bandwidth 8 and gain 125; IR-783: λ_{Ex} = 790 nm and λ_{Em} = 820 nm with bandwidth 13 and gain 175; PTSA: λ_{Ex} = 375 nm and λ_{Em} = 405 nm with bandwidth 5 and gain 150). For each measurement, the plate was read 3 consecutive times to relativize the instrument's influence on the signal.
All obtained fluorescence intensities were background corrected by subtraction of the respective inactivated serum negative control and then normalized to the background corrected fluorescence intensity of the positive control to obtain the liposome lysis. In all cases, gaussian error propagation was used to determine the respective standard deviations.

WILEY-VCH

Supporting Figures

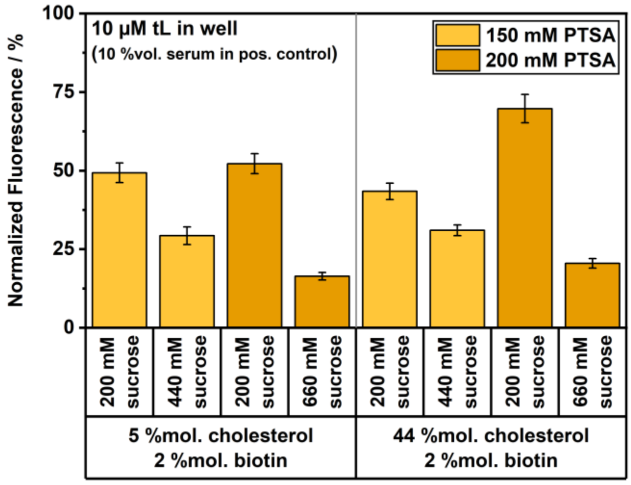

Figure S1. Fluorescence intensities of PTSA encapsulating liposomes (PTSA-Liposome-3, 5, 7 & 8) measured in LCB with varying sucrose content, normalized to a detergent containing positive control (30 mM OG) containing 10 %vol. serum as in liposome complement assays (see materials and methods). Liposomes were measured at 10 µM tL. n=3.

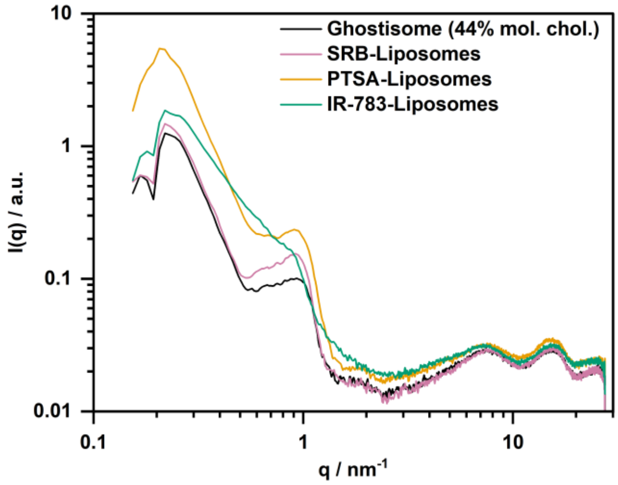

Figure S2. SAXS measurements of fluorophore encapsulating liposomes (44 %mol. cholesterol lipid mixture, ~50 mM total lipid, Ghostisomes-2, SRB-Liposome-4, PTSA-Liposome-2, IR-Liposome-3).

WILEY-VCH

Table S1. Liposome characteristics including lipid composition, encapsulant concentrations in 20 mM HEPES (pH 7.5), smallest membrane pore size during extrusion, Hydrodynamic diameter d(H), polydispersity index PDI, zeta-potential and total lipid concentration tL.

Batch	Lipid composition	Encapsulant & membrane pore size	d(H) / nm	PDI	Zeta potential / mV	tL / mM
**SRB-Liposome-1**	5 %mol. cholesterol, 74 %mol. DPPC, 19 %mol. DPPG 2 %mol. DPPE-biotin	10 mM SRB 210 mM NaCl 0.2 µm	130.1 ± 0.8	0.12 ± 0.01	-16 ± 1	6.26 ± 0.07
**SRB-Liposome-2**	5 %mol. cholesterol, 74 %mol. DPPC, 18 %mol. DPPG 2 %mol. DPPE-biotin 1 %mol. LPS	10 mM SRB 210 mM NaCl 0.4 µm	154 ± 4	0.169 ± 0.007	-8 ± 1	7.22 ± 0.04
**SRB-Liposome-3**	44 %mol. cholesterol, 34 %mol. DPPC, 20 %mol. DPPG 2 %mol. DPPE-biotin	10 mM SRB 210 mM NaCl 0.2 µm	143 ± 2	0.103 ± 0.005	-25 ± 1	9.42 ± 0.03
**SRB-Liposome-4**	44 %mol. cholesterol, 34 %mol. DPPC, 20 %mol. DPPG 2 %mol. DPPE-biotin	10 mM SRB 210 mM NaCl 0.4 µm	275 ± 5	0.17 ± 0.02	-24 ± 2	8.52 ± 0.03
**IR-Liposome-1**	44 %mol. cholesterol, 34 %mol. DPPC, 20 %mol. DPPG 2 %mol. DPPE-biotin	10 mM IR-783 210 mM NaCl 0.4 µm	170 ± 10	0.264 ± 0.006	-23 ± 3	4.45 ± 0.02
**IR-Liposome-2**	44 %mol. cholesterol, 34 %mol. DPPC, 20 %mol. DPPG 2 %mol. DPPE-biotin	10 mM IR-783 210 mM NaCl 0.4 µm	180 ± 6	0.255 ± 0.006	-23 ± 2	8.22 ± 0.04
**IR-Liposome-3**	44 %mol. cholesterol, 34 %mol. DPPC, 19 %mol. DPPG 2 %mol. DPPE-biotin 1 %mol. LPS	10 mM IR-783 210 mM NaCl 0.4 µm	140.7 ± 0.3	0.22 ± 0.01	-6 ± 1	6.6 ± 0.04
**PTSA-Liposome-1**	5 %mol. cholesterol, 73 %mol. DPPC, 20 %mol. DPPG 2 %mol. DPPE-biotin	100 mM PTSA 0.2 µm	136 ± 5	0.089 ± 0.007	-16 ± 1	10.62 ± 0.08
**PTSA-Liposome-2**	44 %mol. cholesterol, 34 %mol. DPPC, 20 %mol. DPPG 2 %mol. DPPE-biotin	100 mM PTSA 0.2 µm	165 ± 7	0.06 ± 0.03	-22 ± 2	7.30 ± 0.03
**PTSA-Liposome-3**	5 %mol. cholesterol, 73 %mol. DPPC, 20 %mol. DPPG 2 %mol. DPPE-biotin	150 mM PTSA 0.2 µm	118 ± 2	0.12 ± 0.03	-19 ± 2	9.12 ± 0.06
**PTSA-Liposome-4**	5 %mol. cholesterol, 74 %mol. DPPC, 20 %mol. DPPG 1 %mol. LPS	150 mM PTSA 0.4 µm	145 ± 4	0.16 ± 0.03	-9 ± 4	3.26 ± 0.01
**PTSA-Liposome-5**	44 %mol. cholesterol, 35 %mol. DPPC, 20 %mol. DPPG 2 %mol. DPPE-biotin	150 mM PTSA 0.2 µm	147.3 ± 0.9	0.08 ± 0.02	-22 ± 2	10.16 ± 0.07
**PTSA-Liposome-6**	44 %mol. cholesterol, 35 %mol. DPPC, 20 %mol. DPPG 1 %mol. LPS 0.4 µm	150 mM PTSA 0.4 µm	176 ± 6	0.21 ± 0.01	-6 ± 2	4.89 ± 0.08

Batch	Lipid composition	Encapsulant & membrane pore size	d(H) / nm	PDI	Zeta potential / mV	tL / mM
**PTSA-Liposome-7**	5 %mol. cholesterol, 73 %mol. DPPC, 20 %mol. DPPG 2 %mol. DPPE-biotin	200 mM PTSA 0.2 µm	141.9 ± 0.4	0.08 ± 0.01	-16 ± 3	10.73 ± 0.08
**PTSA-Liposome-8**	44 %mol. cholesterol, 34 %mol. DPPC, 20 %mol. DPPG 2 %mol. DPPE-biotin	200 mM PTSA 0.2 µm	155 ± 1	0.07 ± 0.02	-18 ± 3	10.80 ± 0.08
**Ghostisome-1**	5 %mol. cholesterol, 73 %mol. DPPC, 20 %mol. DPPG 2 %mol. DPPE-biotin	280 mM NaCl 0.4 µm	220 ± 11	0.20 ± 0.01	-19.1 ± 0.8	11.66 ± 0.05
**Ghostisome-2**	44 %mol. cholesterol, 34 %mol. DPPC, 20 %mol. DPPG 2 %mol. DPPE-biotin	280 mM NaCl 0.4 µm	181 ± 7	0.23 ± 0.02	-25 ± 2	10.3 ± 0.2
**Ghostisome-3**	44 %mol. cholesterol, 34 %mol. DPPC, 20 %mol. DPPG 2 %mol. DPPE-biotin	280 mM NaCl 0.4 µm	301 ± 7	0.16 ± 0.02	-24 ± 2	6.52 ± 0.03

References

[25] K. Hoecherl, S. Streif, C. Spitzenberg, S. Rink, A. Behrent, F. Holzhausen, C. Griesche, C. Rogoll, M. Foedlmeier, A. Gebhard et al., *Analytical and bioanalytical chemistry* **2025**, *417*, 3257.

[51] W. M. Zelek, C. L. Harris, B. P. Morgan, *Immunobiology* **2018**, *223*, 744.

WILEY-VCH
